# Oration on Harvey

**Published:** 1859-11

**Authors:** 


ORATION ON HARVEY.—This oration, instituted many years ago by the College of Physicians of London, is still annually delivered in Latin, that illustrious body being evidently unable to extricate itself from a habit under which it has so long labored. The address, this year, was delivered on the 29th of June, by Dr. C. J. B. Aldis, and is represented as a highly finished production, distinguished by the grace and purity of its style. It strikes us on this side of the waters as very singular that so grave a set of men should make themselves so ridiculous by meeting, once a year, to listen to what very few, we suppose, really only partially comprehend, or which, if fully comprehended, could certainly be much better expressed in good classical English. We agree with our cotemporary, the London Lancet, that this custom would be honored more in the breach than in the observance, and that it shows anything but an evidence of progress.

